# Overexpressing S100A9 ameliorates NK cell dysfunction in estrogen receptor-positive breast cancer

**DOI:** 10.1007/s00262-024-03699-1

**Published:** 2024-05-07

**Authors:** Yansong Liu, Mingcui Li, Zhengbo Fang, Shan Gao, Weilun Cheng, Yunqiang Duan, Xuelian Wang, Jianyuan Feng, Tianshui Yu, Jiarui Zhang, Ting Wang, Anbang Hu, Hanyu Zhang, Zhiyuan Rong, Suborna S. Shakila, Yuhang Shang, Fanjing Kong, Jiangwei Liu, Yanling Li, Fei Ma

**Affiliations:** 1 Department of General Surgery, The Second Affiliated Hospital of Harbin Medical University, 246 Xuefu Street, Harbin, 150001 China; 2 Department of Pathology, The Second Affiliated Hospital of Harbin Medical University, 246 Xuefu Street, Harbin, 150001 China

**Keywords:** S100A9, NK cell, Tumor microenvironment, Breast cancer

## Abstract

**Background:**

Estrogen receptor (ER) positive human epidermal growth factor receptor 2 (HER2) negative breast cancer (ER+/HER2−BC) and triple-negative breast cancer (TNBC) are two distinct breast cancer molecular subtypes, especially in tumor immune microenvironment (TIME). The TIME of TNBC is considered to be more inflammatory than that of ER+/HER2−BC. Natural killer (NK) cells are innate lymphocytes that play an important role of tumor eradication in TME. However, studies focusing on the different cell states of NK cells in breast cancer subtypes are still inadequate.

**Methods:**

In this study, single-cell mRNA sequencing (scRNA-seq) and bulk mRNA sequencing data from ER+/HER2−BC and TNBC were analyzed. Key regulator of NK cell suppression in ER+/HER2−BC, S100A9, was quantified by qPCR and ELISA in MCF-7, T47D, MDA-MB-468 and MDA-MB-231 cell lines. The prognosis predictability of S100A9 and NK activation markers was evaluated by Kaplan–Meier analyses using TCGA-BRAC data. The phenotype changes of NK cells in ER+/HER2−BC after overexpressing S100A9 in cancer cells were evaluated by the production levels of IFN-gamma, perforin and granzyme B and cytotoxicity assay.

**Results:**

By analyzing scRNA-seq data, we found that multiple genes involved in cellular stress response were upregulated in ER+/HER2−BC compared with TNBC. Moreover, TLR regulation pathway was significantly enriched using differentially expressed genes (DEGs) from comparing the transcriptome data of ER+/HER2−BC and TNBC cancer cells, and NK cell infiltration high/low groups. Among the DEGs, S100A9 was identified as a key regulator. Patients with higher expression levels of S100A9 and NK cell activation markers had better overall survival. Furthermore, we proved that overexpression of S100A9 in ER+/HER2-cells could improve cocultured NK cell function.

**Conclusion:**

In conclusion, the study we presented demonstrated that NK cells in ER+/HER2−BC were hypofunctional, and S100A9 was an important regulator of NK cell function in ER+BC. Our work contributes to elucidate the regulatory networks between cancer cells and NK cells and may provide theoretical basis for novel drug development.

**Supplementary Information:**

The online version contains supplementary material available at 10.1007/s00262-024-03699-1.

## Introduction

The interactions between tumor cells and their surrounding microenvironment have been investigated extensively in the past decades. The immune components of tumor microenvironment (TME) include various kinds of immune cells, cytokines and chemokines that contribute to tumor regulation. Depending on the heterogeneity of different cancer types, immune cells can exert both tumor-enabling or suppressing functions in TME [1].

Lymphocytes are comprised of three major cell groups, T cells, B cells and natural killer (NK) cells. Each cell type can be further categorized into subsets based on cell states and molecular expressions. Lymphocytes are known effectors in cellular and humoral anti-tumor immune responses. Among them, cytotoxic lymphocytes, including CD8+T cells, NK cells and γδ T cells, act as direct tumor-killing cells [2]. CD8+T cells require to be activated to perform anti-tumor ability, which involves the participation of MHC-I complexes, costimulatory molecules and various cytokines. To evade clearance by CD8+T cells, tumor cells often display reduced levels of MHC class I antigen presentation-related genes [3]. Whereas, NK cells do not depend on MHC molecules to recognize tumor cells, which are complementary to CD8+functions. Therapeutics that activate NK cells have the potential to be promising next-generation approaches of cancer immunotherapy [4].

There are four molecular subtypes (Luminal A, Luminal B, Her2 and triple negative) of breast cancer (BC), determined by the expression of estrogen receptor (ER), progesterone receptor (PR), human epidermal growth factor receptor 2 (Her2) and Ki-67. Importantly, the response to immunotherapy varied significantly between subtypes, possibly due to the diversity and complexity of tumor immune microenvironment (TIME) [5]. Currently, immunotherapy in triple-negative breast cancer (TNBC) is considered to have the best curative efficacy, leading the approval of immunotherapy for TNBC in clinical settings [5]. Compared with TNBC, the TIME of ER positive BC features fewer tumor-infiltrating lymphocytes (TILs) and lower MHC-I expression level [6]. Interestingly, despite the fact that low MHC-I expression causes NK cell activation, ER+breast cancer cell still escapes from NK cell by upregulating inhibitory receptors and secreting modulatory factors [7]. Moreover, estrogen-associated signaling pathways were found to suppress lymphocytes functions, including NK cells [8, 9]. Understanding the complexity of NK cell regulation, especially subtype-specific mechanisms may facilitate the development of more targeted immunotherapies.

The S100 protein family is composed of 21 multifunctional Calcium-binding proteins, which exist in a wide spectrum of tissues. Despite the similarities in structure, the S100 proteins are involved in distinguished biological processes, such as differentiation and inflammation. The s100 proteins can be secreted by a large variety of living cells, and interact with reciprocal cell surface receptors to fulfill their regulating functions [10]. The S100A9 protein, a member of the S100 family, mainly functions as an immune-modulating factor under pathological conditions. During pathogen invasion, S100A9 is able to promote immune response to defend the host. S100A9 restricts the activity of reverse transcriptase enzymatic, thereby limiting virus infection. Nonetheless, S100A9 was found to be a double-edge sword in anticancer immunity [11]. High expression of S100A9 favors M2 polarization of macrophages, resulting in cancer progression. Conversely, S100A9 can also interact with RAGE receptors and activate NK cells to restrain tumor development in pancreatic cancer [12, 13].

Herein, we presented a study focusing on exploring the possible mechanism behind NK suppression in ER positive breast cancer. By performing in silicon analysis, we noticed that the NK cell-activating state was inferior in ER+/HER2−BC compared with TNBC. After conducting enrichment analysis, we identified that NK cell deactivation could be induced by insufficiency of S100A9 protein. We then validated our findings using in vitro experiments and demonstrated that upregulating S100A9 improved NK cell functions in ER positive breast cancer.

## Methods

### Dataset

Single-cell RNA sequencing data were acquired from the Gene Expression Omnibus (GEO, acquisition number: GSE176078, https://www.ncbi.nlm.nih.gov/geo/query/acc.cgi?acc=GSE176078). TCGA-BRCA mRNA expression datasets and corresponding clinical data were downloaded from TCGA GDC (https://gdc.cancer.gov/) [14, 15]. SCAN-B mRNA expression datasets and corresponding clinical data were retrieved from GSE96058 and GSE81538. The data regarding TNBC subtypes were obtained from Jiang et al. [16].

### Single-cell RNA sequencing analysis

Datasets were combined using R package Seurat (Version: 4.2.0), following the standard Seurat integration workflows [17]. We chose to stick to the cell cluster annotations in the original publication, and NK cell transcriptomes were extracted from ER+/HER2- and TNBC samples [15]. To assess the different expression patterns between the two subtypes, we applied differential analysis, adopting a pseudo-bulk method. Briefly, the gene expression values were pooled per sample for every cell type, and used as the input data for DESeq2 (Version: 1.35.0) [18]. Cutoff values for significant differentially expressed genes (DEGs) were fold change > 1.5 and *P*-value < 0.05 [19]. Cell–cell communications were computed by R package CellChat (Version: 1.6.1). CellChat contains a database of receptor–ligand interactions, and the ligand–receptor pairs between NK cells and other cell types were visualized utilizing the package’s built-in functions [20]. Cell cycle scores of NK cells in each subtype were computed using the CellCycleScoring function from Seurat.

### Bulk RNA sequencing analysis

Immune cell composition for bulk RNA-seq data from TCGA-BRCA and SCAN-B was calculated by a deconvolution algorithm using R package CIBERSORT (Version: 0.1.0) [21] and the xCell algorithm [22]. Next, samples were ordered by activated NK cell proportion (measured by CIBERSORT), and the first and last 25% of samples were selected for differential analysis. Genes varied between groups were identified by Deseq2 with the same criteria in single-cell analysis. Gene ontology (GO) and Reactome enrichment analyses were performed on DEGs by R package ClusterProfiler (Version: 4.3.4) and ReactomePA (Version: 1.39.0), respectively [23, 24]. The expression correlations between S100A9 and NK cell-activating receptors were calculated and plotted in bc-GenExMiner v5.0 database (http://bcgenex.ico.unicancer.fr/) [25]. Expression levels of NK cell activation markers were also acquired from bc-GenExMiner. For transcription factor (TF) and hub genes analyses of DEGs from S100A9-high/ low groups, TFs were downloaded from The Human Transcription Factors website (http://humantfs.ccbr.utoronto.ca/download.php) [26]. The interactions of DEGs were acquired from the STRING database (https://cn.string-db.org/) and visualized in Cytoscape (3.10.1). The hub genes were calculated by cytoHubba using the MCC method with default parameters [27].

### Cell culture

The human hormone receptor (HR) positive breast cancer cell line MCF-7, T47D, human TNBC cell line MDA-MB-231, MDA-MB-468 and human NK cell line NK-92 were purchased from American Type Culture Collection (ATCC). BC cell lines, MCF-7, T47D, MDA-MB-468 and MDA-MB-231, were maintained in DMEM (Thermo Fisher Scientific, Inc.) supplemented with 10% FBS (Thermo Fisher Scientific, Inc.), and 100 mg/ml of penicillin/streptomycin (Thermo Fisher Scientific, Inc.). The NK-92 cell line was maintained in RPMI-1640 (Thermo Fisher Scientific, Inc.) supplemented with 10% FBS, 100 mg/ml penicillin/streptomycin and 100 U/ml interleukin-2 (Thermo Fisher Scientific, Inc.). All cells were incubated at 5% CO2, 37 °C.

### Immunohistochemical staining (IHC)

A total of 151 BC tissues and 45 normal breast samples were obtained from the Department of Breast Surgery, The Second Affiliated Hospital of Harbin Medical University (246 Xuefu Street, Nangang District, Harbin, China). Written informed consent was acquired from all patients who participated in this study, and the research was approved by the Ethics Committee of Harbin Medical University. Formalin-fixed breast tissues were processed into paraffin blocks, and cut into 3-mm-thick serial sections. Hematoxylin and eosin (H&E) staining was used for histologic validation of tissue types. Tissue cores were collected from the paraffin blocks and assembled in an array fashion. The criteria for ER and PR positivity followed the 2010 American Society of Clinical Oncology (ASCO)/College of American Pathologists (CAP) guidelines, and ER/PR positivity was defined as ≥ 1%. The HER2-positive criteria referred to the 2018 ASCO/CAP guidelines, the HER2-positive definitions were: ① IHC 3 + : > 10% of invasive carcinoma cells show strong, complete cell membrane staining. ② IHC 2 + : > 10% of invasive cancer cells show weak to moderate intensity of cell membrane staining or ≤ 10% of invasive cancer cells exhibit strong and complete cell membrane staining, which requires further confirmation using fluorescence in situ hybridization (FISH). Ki-67 score was reported by counting the percentage of positively stained cells in the hotspot areas out of the total number of cells in the field under the microscope. All antibodies used in breast cancer molecular subtyping were purchased from Maixin (Maixin biotechnologies). To perform IHC, tissue microarray was deparaffinized and rehydrated, and subjected to EDTA-mediated high-temperature antigen retrieval. The samples were incubated overnight at 4 °C with the primary anti-S100A9 (Proteintech) and anti-CD16, respectively. After washed in PBS twice, slides were incubated with secondary antibodies for 30 min at room temperature and washed again before stained with DAB. The final expression score of S100A9 was reported using the immunoreactive score (IRS) scoring method, which combined the distribution percentage and intensity scores. The distribution was evaluated as none (0), ≤ 10% (1), 10–25% (2), 25–50% (3) and > 50% (4). Intensity was evaluated as none (0), faint (1), moderate (2) and strong (3). Final expression score = percentage of positive cells multiplied by the staining intensity. The results ranged from 0 to 12. For CD16 positive cells quantitation, the numbers of CD16+cells were counted manually, and the results were described as the number of positive cells per field. All results were reviewed by two pathologists independently.

### Quantitative real-time PCR assay

The expression level of S100A9 in breast cancer cells was evaluated by quantitative real-time PCR (RT-qPCR) assay. Total RNA was extracted using TRIzol reagent (Life Technologies) according to the manufacturer's protocol. The PrimeScript® RT reagent kit (TaKaRa) was used in cDNA reverse transcription. RT-PCR analysis was performed on a 7500 real-time PCR system with SYBR Premix Ex Taq™ Kit (TaKaRa). Primers of S100A9 were bought from Sino Biological (Cat number: HP100072). After completion of the reaction, relative gene expression levels were calculated using the 2 − ΔΔCT method, with β-actin and GAPDH selected as the internal controls. Primers of the internal controls were purchased from OriGene Technologies (β-actin cat number: HP204660, GAPDH cat number: HP205798).

### Cell transfection

To achieve overexpression of S100A9 in MCF-7 and T47D cells, breast cancer cells were cultured in a 24-well plate and transfected with S100A9-expressing (pCMV6-S100A9-Myc-DDK) or empty pCMV6-entry vector (OriGene Technologies, Inc.) using Lipofectamine 2000 (Invitrogen; Thermo Fisher Scientific, Inc.) and DMEM (Thermo Fisher Scientific, Inc.) at 37˚C. After 48 h, stable clones were selected using G-418, and RT-qPCR was performed to confirm the expression level.

### ELISA

To investigate NK cell activity, we assessed the protein levels of IFN-gamma, granzyme B and perforin using ELISA assays. Briefly, NK cells were cocultured with MCF-7/T47D-S100A9-overexpressing and MCF-7/T47D-empty vector cells (ratio: 2:1) in 48-well plates at 37 °C, for 24 h. The supernatant of the coculture systems was collected to measure cytokine secretion. For secretory S100A9 detection, ELISA assays were carried out using the supernatant of MCF-7, T47D, MDA-MB-468 and MDA-MB-231 culture systems. The ELISA kits were purchased from Abcam (IFN-gamma, granzyme B and perforin) and Novus Biologicals (S100A9). The assays were performed following manufactures’ manuals. The optical density (OD) was measured at 450 nm.

### NK cell cytotoxicity assay

MCF-7 and T47D cells were cocultured with NK-92 cells at a ratio of 1:2 in 96-well plates for 48 h at 37 °C, and then the supernatant was collected for lactate dehydrogenase (LDH) assay following the manufacturer's instructions (CyQUANT™ LDH Cytotoxicity Assay, Invitrogen). The absorbance was measured at 490 nm and 680 nm, and the killing function of NK cells was presented as percentage (%) cytotoxicity.

### Statistical analysis

Statistical analyses were done using R software (version 4.2.0) and GraphPad Prism v9.00 (GraphPad Software Inc.). Quantitative data were tested for normal distribution and variance homogeneity using Kolmogorov Smirnova (K-S) method. Differences between groups were analyzed using the Wilcox test or t-test according to the results of K-S test. Categorical variables were assessed using the χ2 test. The Kaplan–Meier analyses were done by the R package survival (3.5–7), and the cutoff values for patient stratification were determined by the function surv_cutpoint. *P* values less than 0.05 were considered statistically significant.

## Results

### Natural killer cells exhibit different states in estrogen receptor-positive breast cancer and triple-negative breast cancer

**Fig. 1:**
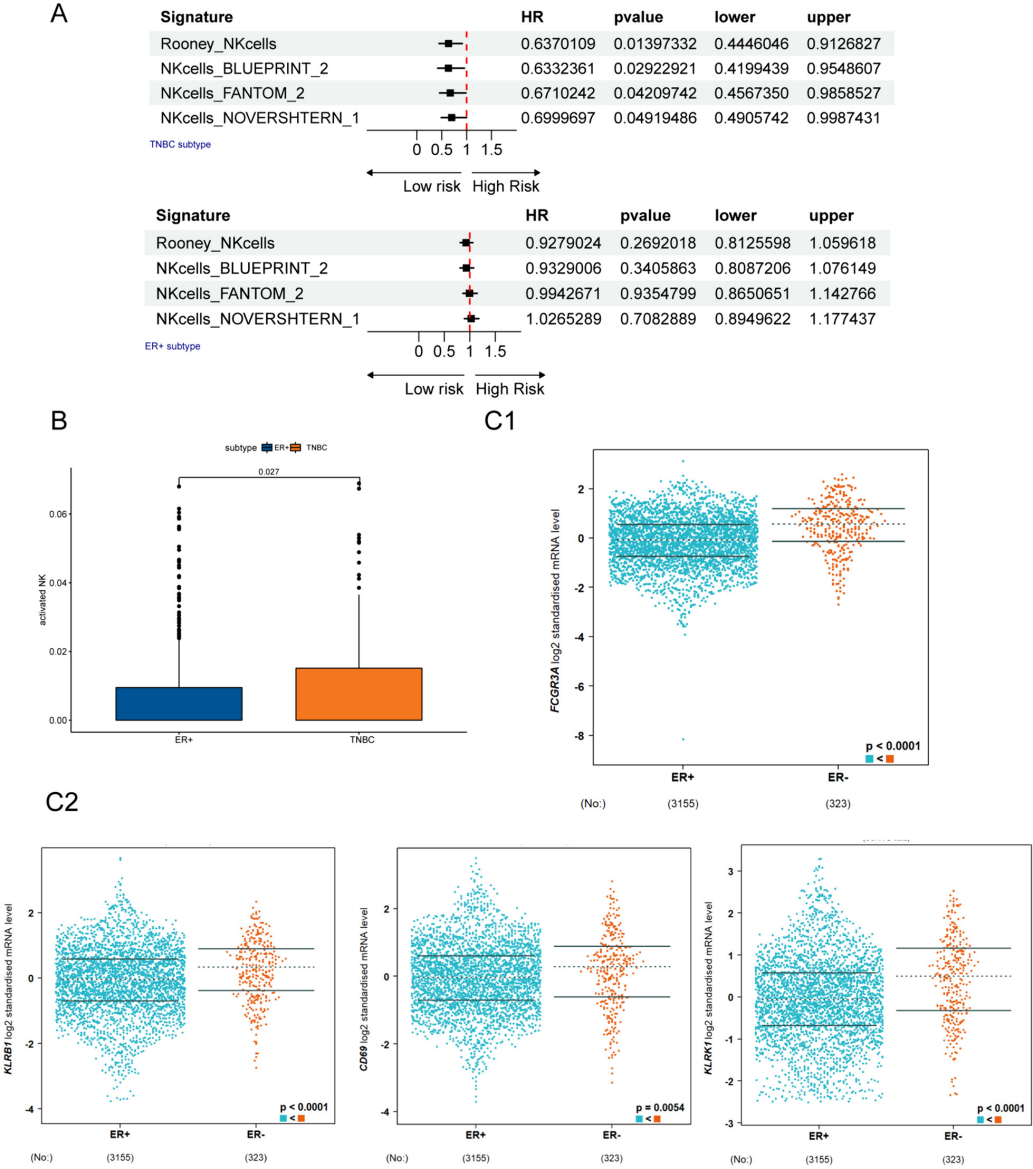
Bulk RNA-seq analyses revealed the difference of NK cells between ER+/HER2−BC and TNBC. **A** Forest plots of univariate cox regression analyses of NK cell-based signature scores calculated by ssGSEA on patients’ survivals. Upper and lower plots show the results of TNBC and ER+/HER2−BC, respectively. The red dash line represents hazard ratio equals one. **B** Immune cell infiltration analysis performed by CIBERSORT indicates that TNBC (right) has more activated NK cells compared to ER+/HER2−BC (left) (C1-2). The mRNA expression levels of NK cell-activating genes (FCGR3A, CD69, KLRB1 and KLRK1) are associated with ER status. *NK* natural killer; *ER* estrogen receptor; *TNBC* triple-negative breast cancer. *P < 0.05, **P < 0.01, ***P < 0.001, ****P < 0.0001

Inflammatory cells are important components of TME and often associated with patients’ survival. To investigate the different prognosis predictability of immune cell-based signatures in ER+/HER2−BC, HER2+BC and TNBC, we gathered and sorted 350 signatures from previous published studies [22, 28–33]. The signatures were quantified by ssGSEA method using mRNA expression data from SCAN-B cohort, and the scores were z-scaled normalized before using as the input data for univariate cox regression analysis. Interestingly, we found that various NK cell-related signatures were statistically significant protective factors in TNBC, partially significant in HER2+BC. However, such effects were not observed in ER+/HER2−BC (Fig. 1A and Fig. s1b). We hypothesized that such discrepancy was due to different functional states of NK cells. Activated NK cell counts were estimated by CIBERSORT algorithm, and the results showed the highest NK cell activation level in TNBC, lowest in ER+/HER2−BC, while ER+/ER–HER2+BC exhibited intermediate activated NK cell infiltration (Fig. 1B and Fig. s1a). TNBC can be categorized into distinct subtypes based on mRNA expression patterns and multiomics data. Consistently, NK cell activation scores were significantly higher in all TNBC subtypes in contrast with ER+/HER2−BC when comparing ER+/HER2−BC with TNBC subtypes (Fig. s1d). Among the types from both FUSCC and Lehmann’s subtyping systems, immunomodulatory (IM) TNBC featured more activated NK cells, which was consistent with previous studies, proving IM being more inflammatory than other subtypes [16, 34].The NK cell activation markers, CD69, KLRB1, KLRK1 and FCGR3A were downregulated in ER+BC (Fig. 1C1, C2) [35]. Furthermore, in breast cancer molecular subtypes, KLRB1 and KLRK1 were higher in TNBC and ER+/HER2+BC in contrast to ER+/HER2−BC (Fig. s1c). From the findings above, TNBC displayed a superior NK cell activation status, whereas NK cells in ER+/HER2−BC were greatly suppressed. Therefore, we decided to investigate the mechanisms causing the deactivation of NK cells in ER+/HER2−BC by comparing with TNBC.

### ***Single-cell sequencing analyses show NK cells possess higher level of cellular stress in ***ER+***breast cancer***

**Fig. 2:**
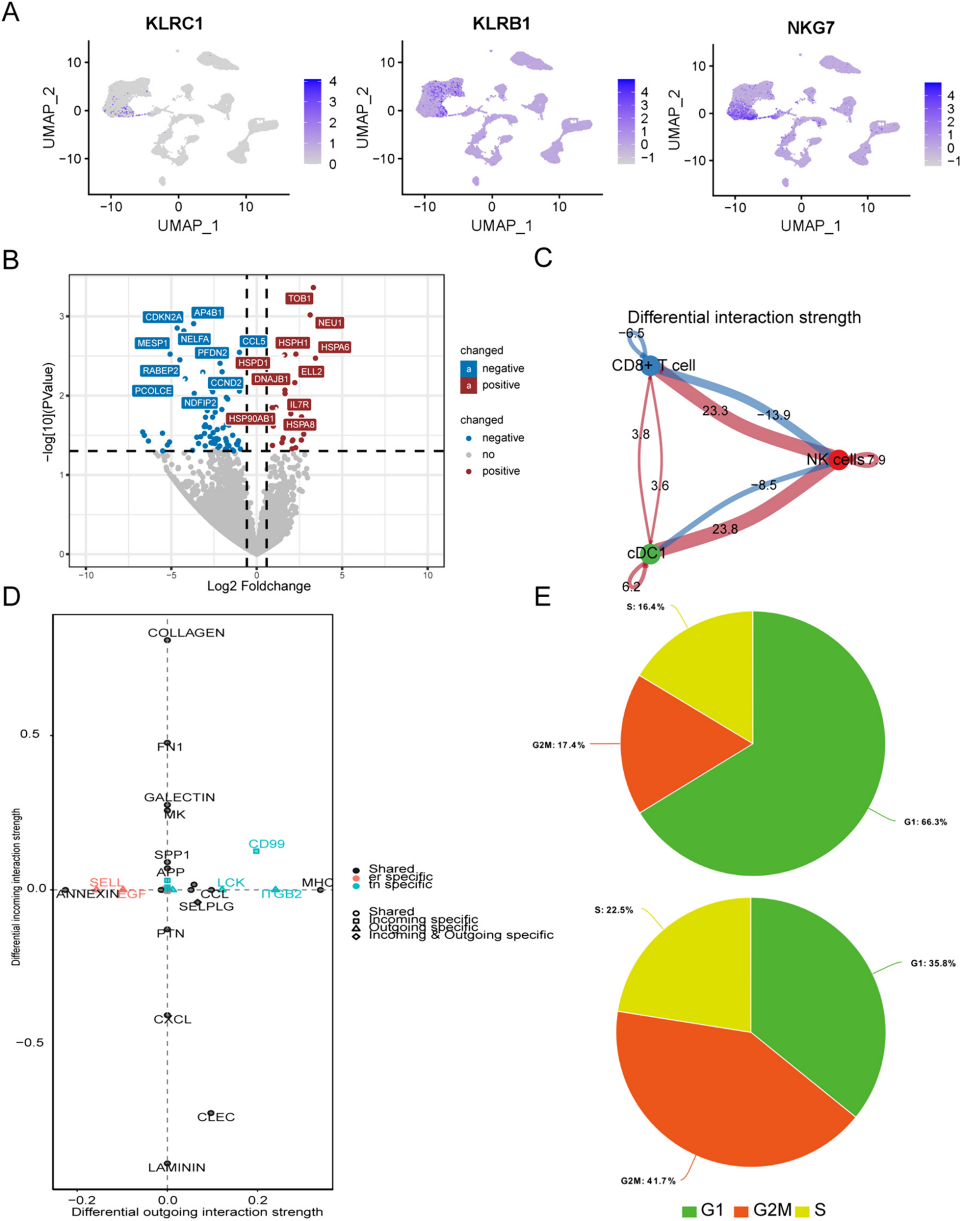
Single-cell RNA-seq analyses revealed the difference of NK cells between ER+/HER2−BC and TNBC. **A** The UMAP plots exhibit the expression levels of NK cell marker genes. **B** Volcano plot of differentially expressed genes between ER+/HER2−BC and TNBC evaluated by pseudo-bulk differential analysis. Red dots represent genes with fold change larger than 1.5 and *p*-value lesser than 0.05. **C** Differential interaction strengths between cell types of ER+/HER2−BC and TNBC are measured by the CellChat algorithm (ER+/HER2−BC as the reference group). Red strings indicate increased interaction strengths. **D** Genes involved in differential interactions are displayed as coordinates, with interactions specific to TNBC colored blue. X-axis and Y-axis represent outgoing interaction strength and incoming interaction strength, respectively (ER+/HER2−BC as the reference group). **E** Pie charts show the cell cycle distributions of ER+/HER2−BC (upper) and TNBC (lower)

To examine NK cell functional states at single-cell resolution, we obtained the scRNA-seq data of ER+/HER2−BC and TNBC samples from GEO database (GSE176078). NK cells were annotated using NK cell markers, KLRC1, KLRB1 and NKG7 (Fig. 2A) [15]. The mRNA expression differential analysis was performed on NK cells from the two molecular subtypes, adopting a pseudo-bulk method. In total, 23 genes were significantly upregulated in ER+/HER2−BC compared to TNBC, whereas 76 genes were downregulated (Table s1). Among the DEGs, the expression levels of various immune-regulating genes were decreased in NK cells from ER+/HER2−BC, including NK activation coreceptor CD59, interleukin-2 receptor IL2RB and interleukin-10 receptor IL10RA, which have been proved to be conducive to NK cell proliferation and effector function [36–38]. Besides, the mRNA expressions of NK cell-producing cytokines CCL4 and CCL5 were also reduced [39]. Interestingly, we noticed that multiple heat shock protein (HSP) family members were upregulated in ER+/HER2−BC NK cells (HSPA6, HSPD1, HSPA8, HSP90AB1 and HSP90AA1) (Fig. 2B). HSPs are essential participants of cellular stress responses, which could be induced by hypoxia and other external or internal stimulus [40]. Tang et al. identified a NK cell cellular stress state, characterized by elevated expression of HSP90AA1, HSP90AB1, DNAJB1, etc., with impaired cytotoxicity [41]. This NK cell stress marker gene set significantly overlapped with the genes upregulated in NK cells originated from ER+/HER2−BC. Since cell stress influences proliferation and triggers cell death, we performed cell cycle analysis [40]. The G2/M phase NK cells showed a higher distribution in TNBC, reflecting suppressed proliferation of NK cells in ER+/HER2−BC (Fig. 2E). Using CellChat algorithm, we found that compared with TNBC, NK cells in ER+/HER2−BC displayed decreased outgoing interaction strengths to CD8 + T cells and cDCs, which may indicate hypofunction (Fig. 2C). Moreover, cell communication analysis presented elevated interaction strength of receptors and cytokines important for NK cell maturation and activation, such as CD99, ITGB2 and CCL in TNBC [42, 43] (Fig. 2D).

### S100 calcium-binding protein A9 potentially regulates natural killer cells in estrogen receptor-positive breast cancer

**Fig. 3:**
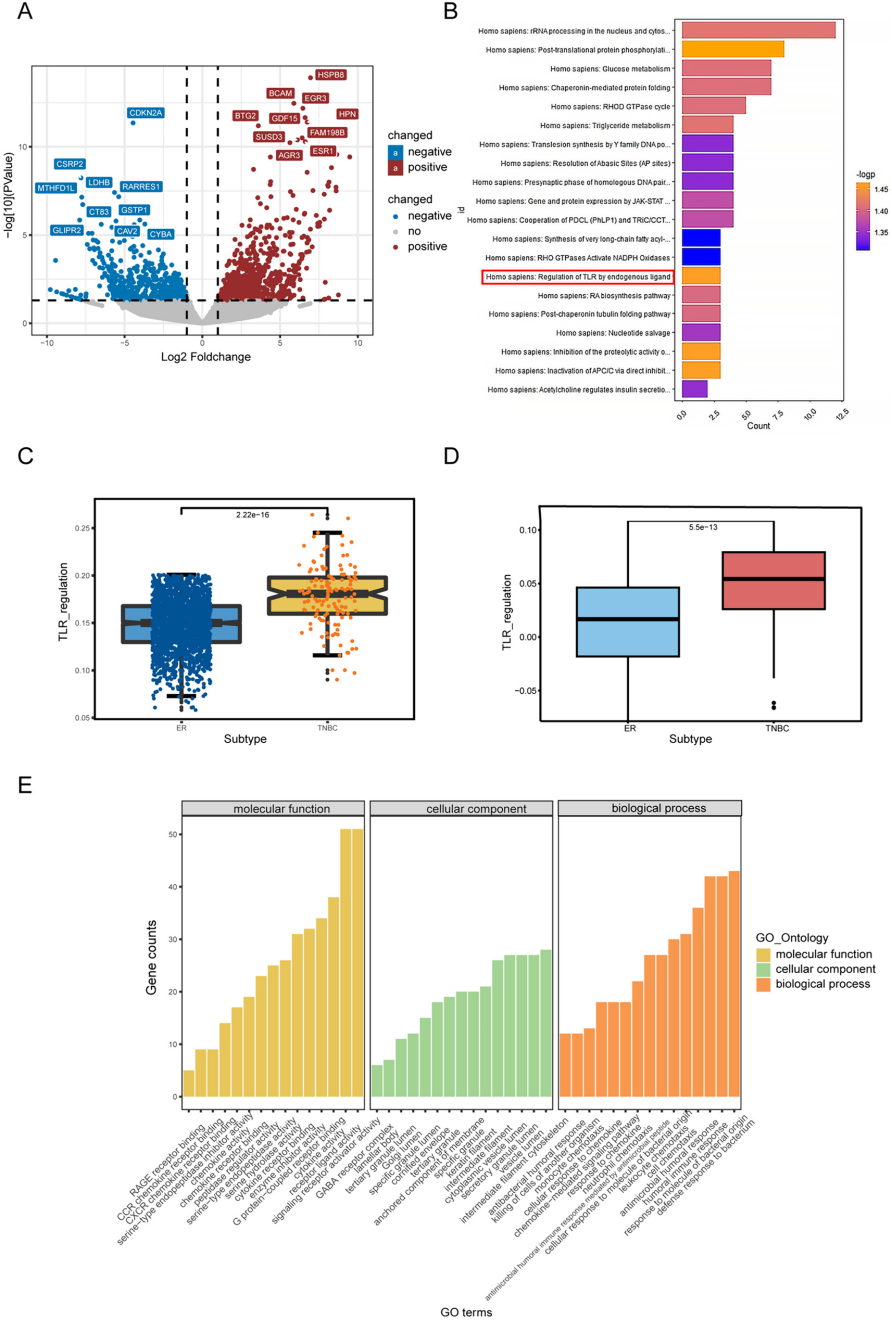
TLR pathway-related gene S100A9 is associated with NK cell hypofunctional states in ER+/HER2−BC. **A** Volcano plot of differential genes (DEGs) between ER+cancer cells and TNBC cells identified by pseudo-bulk differential analysis. **B** Reactome enrichment analysis of DEGs between ER+/HER2−BC and TNBC cells. **C**, **D** Boxplots exhibit the distribution of TLR regulation signature scores of ER+/HER2−BC and TNBC using data from SCAN-B (left) and TCGA (right) cohorts. **E** GO enrichment analysis of DEGs between S100A9-high and S100A9-low ER+/HER2−BC. Abbreviations: TLR, Toll-like receptor; GO, gene ontology

**Fig. 4:**
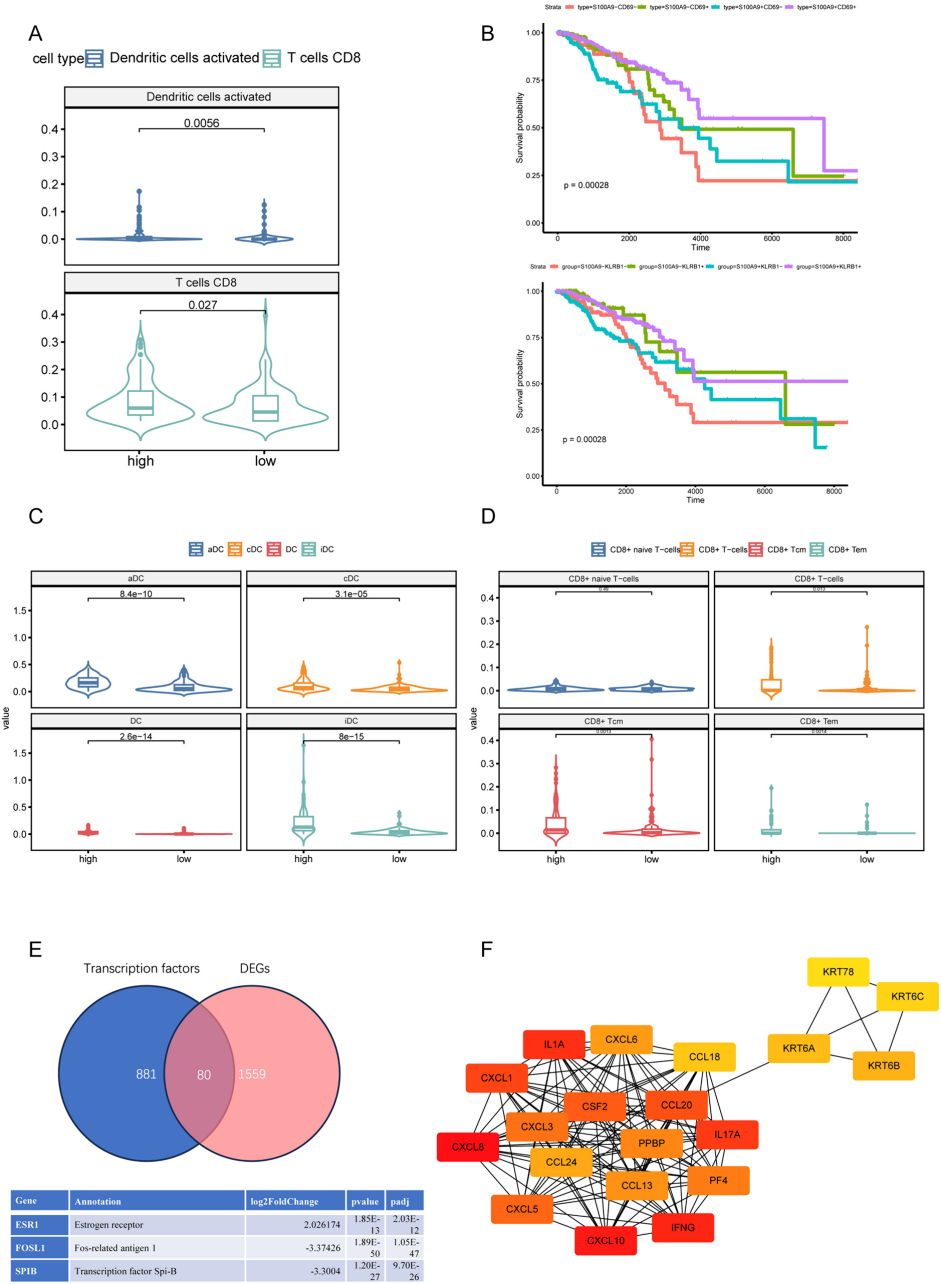
The expression level of S100A9 associates with immune infiltration and prognosis. **A** S100A9-high ER+/HER2−BC (left) shows higher infiltration of NK cell function-related immune cells (CD8 + T cell and dendritic cell) compared to their S100A9-low counterparts (right) calculated by CIBERSORT. **B** Kaplan–Meier curves using TCGA cohort stratified by S100A9 and CD69/KLRB1 expression levels. **C** Violin plots of the infiltration levels of DC cell and its subtypes quantified by xCell algorithm in S100A9-high/low groups using TCGA data. **D** Violin plots of the infiltration levels of CD8 + T cell and its subtypes quantified by xCell algorithm in S100A9-high/low groups with TCGA cohort. **E** Venn plot showing the number of TFs overlapped with DEGs from differential analysis between S100A9-high/low groups. **F** Network of hub genes identified by Cytohubba from DEGs (bright red indicates high MCC score). *TF* transcription factors

Since the cross talk between cancer cells and immune cells plays an indispensable role in shaping TME, we decided to explore the possible regulatory pathways of cancer cells that lead to NK cell functional suppression in ER+/HER2−BC. Utilizing scRNA-seq data, we conducted a differential analysis comparing the transcriptome of ER+/HER2–breast cancer cells and triple-negative breast cancer cells (Fig. 3A). Expectedly, multiple estrogen-dependent genes were upregulated in ER+/HER2−BC, including ESR1, PGR, ERBB4, TFF1, TFF3, etc. The enrichment analysis using gene sets from Reactome database was performed afterward, and the DEGs were significantly enriched in pathways involved in RNA processing, posttranslational modification and TLR regulation (Fig. 3B). To further clarify the possible mechanism for NK cell hypofunction, we also performed differential and enrichment analyses in activated NK cell-high/low patients defined by CIBERSORT scores (Fig. s2a). We identified the regulation of TLR by endogenous ligand as a common pathway, which was also validated by ssGSEA scoring of TLR regulatory genes in ER+/HER2−BC and TNBC using mRNA expression data from the TCGA and SCAN-B cohorts (Fig. 3C, D). The TLR regulation signature was higher in all TNBC subtypes compared with ER+/HER2−BC (Fig. s2c, d). Furthermore, the gene S100A9, which is a participant in TLR regulation, was upregulated in TNBC and all TNBC subtypes (Fig. s3a, s3c). S100A9 showed a stronger correlation with NK cell-activating receptors (FCGR3A, KLRB1, KLRK1, NCR1 and NCR3) in ER+BC compared with TNBC (Fig. s3b) [35]. Using the expression value of S100A9 as a divider, we found that S100A9-high patients (the first quarter of patients ranked by S100A9 expression) had higher infiltration levels of DCs and CD8 + T cells utilizing the CIBERSORT algorithm (Fig. 4A). We further validated this observation with the xCell method for immune infiltration analysis in TCGA-BRCA and SCAN-B cohorts (Fig. 4C, D, Fig. s3d, e). The GO enrichment of DEGs from S100A9-high/low patients showed significant enrichment in immune-related biological processes, such as leukocyte chemotaxis and response to chemokine (Fig. 3E). Furthermore, the hub genes identified by the cytoHubba algorithm were predominately immune-related, including IFNG and various CCL and CXCL chemokines (Fig. 4F). The abnormally expressed transcription factors (TFs) might contribute to S100A9 overexpression. To explore the possible upstream of S100A9 dysregulation, we identified TFs from the DEGs, with ASCL1 and LIN28B being the most upregulated and downregulated TFs compared to S100A9-high group (Fig. 4E, Table s2). S100A9 has been proved to be transcriptionally regulated by PU.1 and AP-1, which was in concordance with our finding that the PU.1-related gene SPIB and AP-1 transcription factor FOSL1 were differentially expressed in S100A9-high group (Table s2) [44, 45]. Notably, ESR1, the estrogen receptor coding gene, was among the overexpressed TFs in S100A9-low group, which further validated that estrogen receptor signaling could contribute to immune suppression [46]. Since NK cell was considered to be associated with better prognosis, we next investigated the impact of S100A9 and NK cell activation markers on patients’ survivals. S100A9 + CD69 + and S100A9 + KLRB1 + patients showed longer overall survivals (Fig. 4B), which was not detected in S100A9 + CD16 + patients (data not shown), possibly due to the mixed effects of CD16 on prognosis [47, 48]. Moreover, after performing survival analyses in molecular subtypes, we found that the prediction ability of S100A9 + CD69 + /KLRB1 + for better prognosis was only significant in ER+/HER2−BC (Fig. s4a–c). To sum up, we discovered S100A9 as a potentially important regulator of NK cell dysfunction in ER+/HER2−BC.

### Overexpression of S100A9 improves natural killer cell functions

**Fig. 5:**
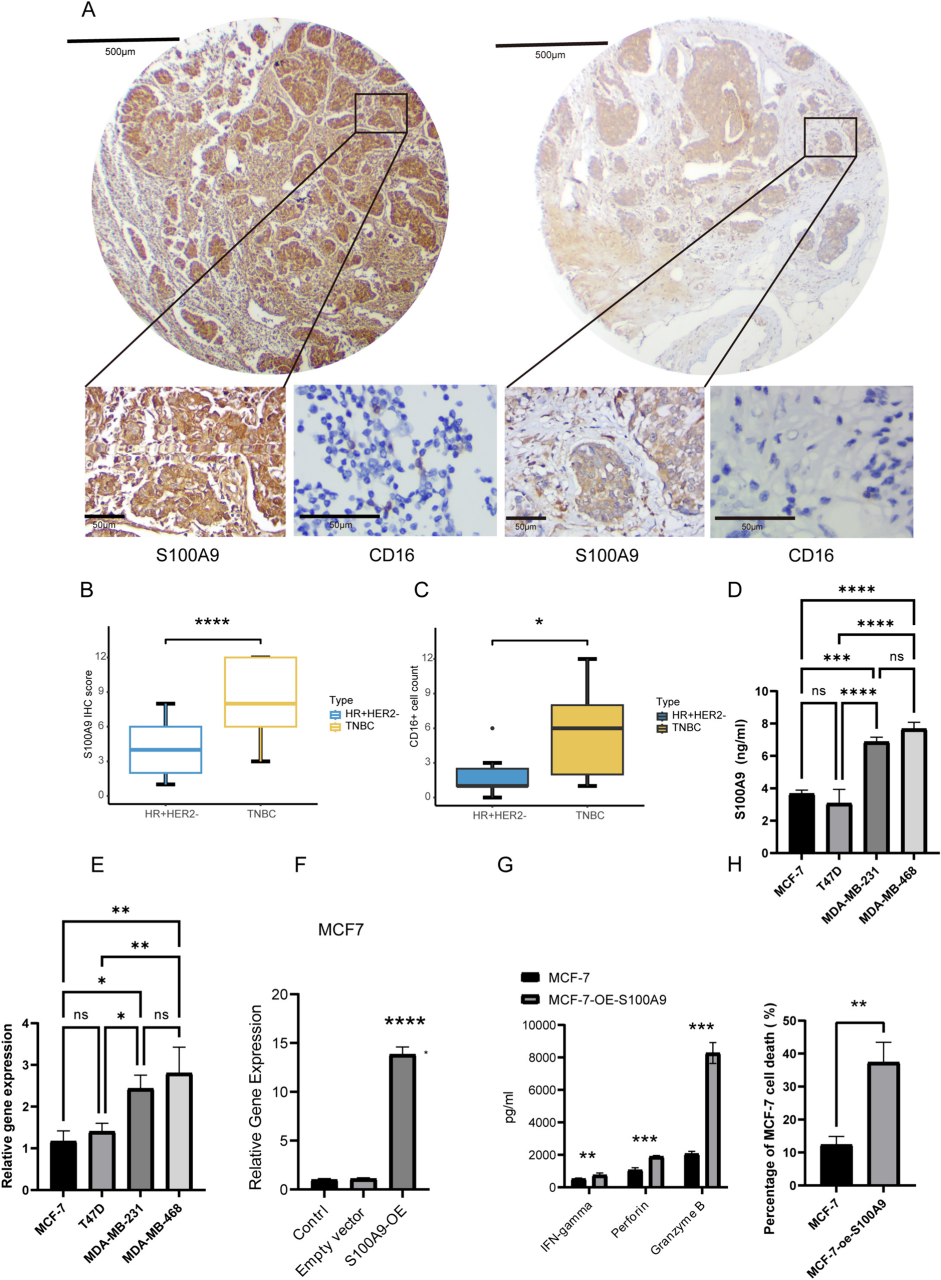
Overexpression of S100A9 enhances natural killer cell functions in ER+/HER2−BC. **A** Representative images of S100A9 (upper and lower left) and CD16 (lower right) stained by IHC in ER+/HER2−BC (right) and TNBC (left). **B** Boxplots of the distribution of S100A9 IHC scores in HR + HER−BC and TNBC. **C** Boxplots display the CD16 positive cell counts in HR + HER−BC and TNBC. **D** ELISA quantities the levels of S100A9 protein in MCF-7, T47D, MDA-MB-231 and MDA-MB-468 cell lines. **E** The qPCR experiment quantities the levels of S100A9 mRNA in MCF-7, T47D, MDA-MB-231 and MDA-MB-468 cell lines. **F** The qPCR experiment validates the S100A9 expression after transfection of S100A9-overexpression vector. **G** ELISA measures the secretion levels of IFN-gamma, perforin and granzyme B from NK cell cocultured with S100A9-overexpressing MCF-7 cell line. **H** LDH NK cell cytotoxicity assay of NK cell cocultured with S100A9-overexpressing MCF-7 cell line. *P < 0.05, **P < 0.01, ***P < 0.001, ****P < 0.0001

Next, we decided to validate the function of S100A9 in vitro. We stained S100A9 and NK cell activation marker CD16 in surgically removed specimens of ER+/HER2−BC and TNBC using IHC. In concordance with our findings in silico, TNBC had higher protein level of S100A9 and more CD16 + cells (Fig. 5A–C). Moreover, the production of S100A9 from breast cancer cells was quantified by ELISA. S100A9 production level was higher in TNBC cell lines (MDA-MB-231 and MDA-MB-468) in contrast with MCF-7 and T47D (Fig. 5D). This finding was also confirmed in mRNA level by qRT-PCR (Fig. 5E). S100A9 was overexpressed in MCF-7 cells by transfection with the S100A9-expressing vector (Fig. 5F). After coculturing with NK-92 cells, we found that overexpressing S100A9 in MCF-7 prominently changed the function of NK cells, enhancing the ability of IFN-gamma, perforin and granzyme B production (Fig. 5G). The cytotoxicity of NK cells cocultured with MCF-7 cells was measured using a lactate dehydrogenase (LDH) cytotoxicity assay. The result revealed that S100A9 overexpression in cancer cells enhanced the cytotoxicity of cocultured NK-92 cells (Fig. 5H). We repeated aforementioned experiments in T47D cells and reached similar conclusions (Fig. s4d–f).

## Discussion

The heterogeneity of tumor immune microenvironment in different BC molecular subtypes has long been an intricate problem to decipher. Elucidating regulatory mechanisms specific to subtypes will contribute to uncovering novel immunotherapy targets, improving patients’ survival. The enhanced expression level of estrogen receptor is involved in immune suppression and has a great impact on treatment strategies [6]. Therefore, comparing ER+/HER2−BC with TNBC might uncover mechanisms inducing suppressive TIME in ER+/HER2−BC, and change the inferior treatment responses of immunotherapy. In this study, we focused on investigating the difference of NK cell function between ER+/HER2−BC and TNBC. We performed multiple bioinformatic analyses and discovered S100A9 as a key factor affecting NK cell status, which was specific to ER+/HER2−BC. By overexpressing S100A9 in ER+breast cancer cells, we demonstrated that upregulating S100A9 could improve cocultured NK cell function. Receptors of S100A9, including TLR4, RAGE, CD147, MCAM and NPTN are expressed in multiple cell types, such as cancer cells, MDSCs, macrophages and NK cells. By interacting with these receptors, S100A9 activates downstream signaling pathways in the target cells, influencing the development of cancer [11, 13]. However, since S100A9 originates from various cell types and plays contradict roles in different cell–cell interactions, more studies need to be done to understand the immune-modulatory function of S100A9 [49]. Nowadays, there has been ongoing trials based on TLR-related immunotherapies [50]. Whether S100A9, the TLR regulator, can be an exploitable drug target remains to be further studied.

Cytotoxic cells fail to eradicate cancer cells in TME due to diverse internal or external signals induced dysfunction [51]. There are several dysfunctional states of NK cells in TME, mainly exhaustion, anergy and senescence. T cell exhaustion, first studied in CD8 + T cell, is characterized by continuous loss of T cell function and upregulation of inhibitory receptors during cancer or chronic infection. Exhausted T cells can be divided into progenitor exhausted and terminal exhausted, and the terminal exhausted T cells show limited respond to anti-PD-1 therapy [52]. Like T cells, NK cells can exhibit exhausted traits when experienced chronic antigen stimulation. Exhausted NK cells show decreased ability of cytokine production, reduced Ki-67 expression and imbalanced distribution of activating and inhibitory receptors. Nonetheless, unlike T cell exhaustion, which is extensively studied, no consensus has been reached for NK cell exhaustion [53]. Anergy, induced by insufficient activating signal (adaptive tolerance) or inadequate costimulation (clonal anergy), leads to impaired proliferation and cytokine production and serves to periphery tolerance, protecting the host from autoimmune disease [51]. In TME, lack of requirements for maximal activation, such as CD137 signaling, may cause NK cell anergy [54]. However, little direct evidence has been obtained to support NK cell anergy in TME till this day. Senescence, characterized by telomere shortenings and cell cycle arrest, has been observed in T cells cocultured with cancer cells [55]. As for NK cells, senescence in TME has not been well described yet, partly due to the debating life span of NK cells [54]. By analyzing single-cell atlas of T cells from 308,048 transcriptomes, Chu et al. defined a T cell stress response state in TME and proved its role in immunotherapy resistant [56]. Furthermore, Tang et al. found the similar state in NK cells [41]. Targeting T cell stress might provide a new treatment option for cancer immunotherapy.

The biological functions of NK cells in breast cancer mainly include cancer cell eradication and cytokine production, which suggests that NK cell may associate with favorable prognosis [7]. By assessing the mRNA expression levels of NK cell-activating receptors, Ascierto et al. demonstrated that the NK cell signature related to better prognosis in BC patients [57]. However, NK cells feature various differentiate and functional states in the cancer TIME. Studies have found that increased level of CD56 expression was related to impaired cytotoxicity, while CD16 positivity indicated cell killing ability [58]. A study performed by Thacker et al. found that CD56^bright^ NK cells correlated with worse survival in TNBC [59]. Nonetheless, Bouzidi et al. performed a study using CD56 as a marker for NK cell infiltration and stained patient-derived breast cancer tissues with IHC method. They proved that CD56 expression could predict prolonged overall survival, regardless of the molecular subtypes [60]. This inconsistency might be caused by different detection methods for NK cells, or by the expression of CD56 on NK cells with contradict functions since CD56 + NK cells can be further divided into subclusters [58]. Therefore, before applying NK cells as a prognosis indicator in clinical settings, more work has to be done to elucidate the biological functions of NK cell subtypes, and select the appropriate markers and quantification methods for NK cells.

In conclusion, our study found that NK cells in ER+/HER2−BC exhibited a hypofunctional state, and overexpressing S100A9 in cancer cells improved NK cells function in ER+/HER2−BC. Our work contributes to clarify the regulatory networks between cancer cells and NK cells and may provide theoretical basis for novel drug development.

### Supplementary Information

Below is the link to the electronic supplementary material.

Supplementary file1 (DOCX 1386 KB)

## Data Availability

Please contact the corresponding authors for all data requests.
