# Supplementary material for: Overexpressing S100A9 ameliorates NK cell dysfunction in estrogen receptor-positive breast cancer
Source: Cancer Immunol Immunother. 2024 May 7;73(7):117. doi: 10.1007/s00262-024-03699-1 (PMC11076447; doi:10.1007/s00262-024-03699-1)
Supplement: Supplementary file 1 — Supplementary file1 (DOCX 1386 KB) [file 262_2024_3699_MOESM1_ESM.docx]

| Gene symbol | log2FoldChange | p-value | changed |
| --- | --- | --- | --- |
| SMAP2 | 1.995017 | 0.016938 | positive |
| HSPA6 | 3.437891 | 0.003373 | positive |
| UAP1 | 2.06898 | 0.046872 | positive |
| HSPD1 | 1.639845 | 0.003065 | positive |
| IL7R | 1.657659 | 0.009425 | positive |
| SSBP2 | 2.747848 | 0.030652 | positive |
| ELL2 | 2.229983 | 0.006859 | positive |
| NEU1 | 3.127651 | 0.00096 | positive |
| HSP90AB1 | 0.906802 | 0.014177 | positive |
| SGK1 | 2.613854 | 0.03638 | positive |
| MRPL18 | 1.537562 | 0.0349 | positive |
| ZFAND2A | 2.626449 | 0.018528 | positive |
| AC090498.1 | 1.476945 | 0.039211 | positive |
| DNAJA1 | 0.923342 | 0.042543 | positive |
| IFITM3 | 1.550937 | 0.034116 | positive |
| HSPA8 | 1.121434 | 0.014033 | positive |
| HSPH1 | 2.292556 | 0.002995 | positive |
| HSP90AA1 | 0.970572 | 0.02423 | positive |
| TOB1 | 3.315701 | 0.000433 | positive |
| METRNL | 2.07948 | 0.037264 | positive |
| DNAJB1 | 1.650443 | 0.008512 | positive |
| DEDD2 | 2.115004 | 0.036369 | positive |
| SLC1A5 | 2.275953 | 0.04544 | positive |
| KCNAB2 | -2.42686 | 0.016173 | negative |
| MAD2L2 | -2.16635 | 0.043264 | negative |
| AP4B1 | -3.69192 | 0.001237 | negative |
| WDR3 | -3.50816 | 0.033498 | negative |
| PFDN2 | -2.14451 | 0.003911 | negative |
| ARPC5 | -1.77616 | 0.010543 | negative |
| CNIH4 | -2.57887 | 0.008935 | negative |
| GPR137B | -3.01232 | 0.043101 | negative |
| AC092580.4 | -1.42056 | 0.048392 | negative |
| DPY30 | -1.79907 | 0.047868 | negative |
| HTRA2 | -2.49021 | 0.035403 | negative |
| COL6A3 | -4.18243 | 0.01196 | negative |
| HIGD1A | -2.67304 | 0.039295 | negative |
| NCK1 | -1.77557 | 0.037727 | negative |
| TM4SF1 | -2.98154 | 0.024464 | negative |
| ABCF3 | -3.65741 | 0.009373 | negative |
| NELFA | -4.26305 | 0.001526 | negative |
| S100P | -5.10566 | 0.038464 | negative |
| CXCL13 | -3.28127 | 0.032922 | negative |
| AK6 | -2.35362 | 0.034763 | negative |
| MRPL22 | -2.30492 | 0.026797 | negative |
| NOL7 | -1.75923 | 0.047128 | negative |
| TDP2 | -3.06751 | 0.024579 | negative |
| PSMB9 | -1.17909 | 0.040819 | negative |
| CBX3 | -1.62765 | 0.042911 | negative |
| MPLKIP | -2.67966 | 0.008139 | negative |
| PCOLCE | -4.18469 | 0.006122 | negative |
| CNOT7 | -1.82962 | 0.047231 | negative |
| LY6E | -1.13422 | 0.041663 | negative |
| CDKN2A | -4.65472 | 0.001404 | negative |
| GALT | -2.54874 | 0.0293 | negative |
| SIT1 | -5.51466 | 0.02982 | negative |
| CD59 | -1.708 | 0.041535 | negative |
| UQCC3 | -2.47073 | 0.03712 | negative |
| PRDX5 | -1.77532 | 0.007178 | negative |
| RAB1B | -1.8172 | 0.034893 | negative |
| PPP1CA | -1.62157 | 0.023599 | negative |
| GSTP1 | -0.95275 | 0.047225 | negative |
| LAMTOR1 | -1.77218 | 0.022943 | negative |
| IL10RA | -2.04084 | 0.01496 | negative |
| TYSND1 | -3.40612 | 0.040152 | negative |
| USP54 | -6.67306 | 0.028772 | negative |
| CCND2 | -2.02535 | 0.005002 | negative |
| NELL2 | -2.73086 | 0.032877 | negative |
| NDFIP2 | -3.15272 | 0.005069 | negative |
| MGAT2 | -2.7597 | 0.025215 | negative |
| IGHA1 | -2.24613 | 0.035502 | negative |
| MESP1 | -5.06858 | 0.003001 | negative |
| UBN1 | -2.27755 | 0.04655 | negative |
| CLN3 | -2.58118 | 0.018686 | negative |
| RABEP2 | -4.49824 | 0.003534 | negative |
| TLDC1 | -5.22646 | 0.042003 | negative |
| CPNE7 | -2.89307 | 0.015424 | negative |
| DERL2 | -1.97498 | 0.025579 | negative |
| TRAPPC1 | -1.40874 | 0.031607 | negative |
| CCL5 | -1.01167 | 0.002842 | negative |
| CCL4 | -1.01023 | 0.008766 | negative |
| AATF | -2.99754 | 0.01874 | negative |
| KANSL1-AS1 | -3.75323 | 0.048976 | negative |
| NUP85 | -3.49875 | 0.013294 | negative |
| ANAPC11 | -1.35064 | 0.049925 | negative |
| ADA | -2.38758 | 0.037008 | negative |
| PSMA7 | -1.10172 | 0.039213 | negative |
| TPGS1 | -2.4531 | 0.040196 | negative |
| SGTA | -3.05458 | 0.030003 | negative |
| SNAPC2 | -2.62378 | 0.039655 | negative |
| MYO1F | -1.70019 | 0.011052 | negative |
| ACP5 | -3.00667 | 0.023349 | negative |
| NDUFA13 | -2.20142 | 0.007387 | negative |
| HAMP | -6.5787 | 0.031734 | negative |
| IL2RB | -1.07011 | 0.037175 | negative |
| CPT1B | -6.5787 | 0.031734 | negative |
| RP11-54O7.17 | -6.33765 | 0.037412 | negative |
| IGLV7-46 | -5.50551 | 0.049792 | negative |
| CSN3 | -6.5787 | 0.031734 | negative |
| CSN1S1 | -6.5787 | 0.031734 | negative |

Table S1. Pseudo-bulk differential analysis results of NK cells in ER+ BC and TNBC (TNBC as the reference group)

| Gene symbol | Annotation | log2FoldChange | pvalue | padj |
| --- | --- | --- | --- | --- |
| ASCL1 | Achaete-scute homolog 1 | 3.97871 | 9.13E-14 | 1.07E-12 |
| MYT1 | Myelin transcription factor 1 | 3.208118 | 5.31E-23 | 2.48E-21 |
| ZNF804B | Zinc finger protein 804B. | 3.1313 | 1.07E-07 | 4.65E-07 |
| MYOG | Myogenin | 2.745865 | 1.43E-12 | 1.38E-11 |
| RFX6 | DNA-binding protein RFX6 | 2.646566 | 4.09E-08 | 1.91E-07 |
| INSM1 | Insulinoma-associated protein 1 | 2.545663 | 1.71E-09 | 1.00E-08 |
| DLX2 | Homeobox protein DLX-2 | 2.031101 | 3.08E-08 | 1.47E-07 |
| ESR1 | Estrogen receptor | 2.026174 | 1.85E-13 | 2.03E-12 |
| BCL11A | B-cell lymphoma/leukemia 11A | -2.00798 | 6.58E-12 | 5.71E-11 |
| HMGA2 | High mobility group AT-hook 2. | -2.02216 | 9.87E-16 | 1.59E-14 |
| NR1H4 | Bile acid receptor | -2.02589 | 0.000375 | 0.000879 |
| VSX2 | Visual system homeobox 2 | -2.03511 | 2.56E-07 | 1.04E-06 |
| SOX14 | Transcription factor SOX-14 | -2.0426 | 0.002914 | 0.005757 |
| SOX10 | Transcription factor SOX-10 | -2.0478 | 1.01E-06 | 3.73E-06 |
| HELT | Hairy and enhancer of split-related protein HELT | -2.06104 | 0.010493 | 0.018373 |
| MYOD1 | Myoblast determination protein 1 | -2.06476 | 0.000368 | 0.000863 |
| FOXL2 | Forkhead box protein L2 | -2.07494 | 3.49E-12 | 3.18E-11 |
| LHX8 | LIM/homeobox protein Lhx8 | -2.08623 | 9.25E-09 | 4.80E-08 |
| BARHL2 | BarH-like 2 homeobox protein | -2.10118 | 7.14E-05 | 0.00019 |
| LTF | Lactotransferrin | -2.12813 | 9.87E-10 | 6.02E-09 |
| SOX2 | Transcription factor SOX-2 | -2.13187 | 5.18E-07 | 2.01E-06 |
| ZIC4 | Zinc finger protein ZIC 4 | -2.14999 | 5.98E-09 | 3.21E-08 |
| CBX2 | Chromobox protein homolog 2 | -2.16613 | 1.81E-26 | 1.25E-24 |
| GBX2 | Homeobox protein GBX-2 | -2.17791 | 4.61E-13 | 4.80E-12 |
| RFX4 | Transcription factor RFX4 | -2.19185 | 4.15E-08 | 1.94E-07 |
| GSC2 | Homeobox protein goosecoid-2 | -2.20102 | 2.86E-09 | 1.62E-08 |
| ZFP57 | Zinc finger protein 57 homolog | -2.21367 | 2.70E-09 | 1.54E-08 |
| FOXQ1 | Forkhead box protein Q1 | -2.25869 | 5.32E-18 | 1.23E-16 |
| HOXD11 | Homeobox protein Hox-D11 | -2.31776 | 1.78E-14 | 2.34E-13 |
| ARNTL2 | Aryl hydrocarbon receptor nuclear translocator-like protein 2 | -2.36966 | 2.94E-48 | 1.41E-45 |
| ZBED2 | Zinc finger BED-type containing 2. | -2.37481 | 2.09E-17 | 4.35E-16 |
| LHX2 | LIM/homeobox protein Lhx2 | -2.39672 | 9.08E-14 | 1.07E-12 |
| EN1 | Homeobox protein engrailed-1 | -2.40201 | 2.08E-14 | 2.71E-13 |
| MSGN1 | Mesogenin-1 | -2.40451 | 6.60E-08 | 2.98E-07 |
| ALX1 | ALX homeobox protein 1 | -2.41964 | 3.33E-09 | 1.86E-08 |
| IRX1 | Iroquois-class homeodomain protein IRX-1 | -2.44236 | 1.16E-14 | 1.58E-13 |
| FOXC1 | Forkhead box protein C1 | -2.50208 | 2.13E-25 | 1.32E-23 |
| HOXA11 | Homeobox protein Hox-A11 | -2.52209 | 6.59E-23 | 3.02E-21 |
| ZIC1 | Zinc finger protein ZIC 1 | -2.54691 | 1.10E-09 | 6.69E-09 |
| POU6F2 | POU domain, class 6, transcription factor 2 | -2.5526 | 1.27E-10 | 8.97E-10 |
| HOXD12 | Homeobox protein Hox-D12 | -2.57994 | 1.13E-07 | 4.91E-07 |
| PAX6 | Paired box protein Pax-6 | -2.61035 | 1.84E-13 | 2.03E-12 |
| ETV3L | ETS translocation variant 3-like protein | -2.74176 | 2.03E-12 | 1.91E-11 |
| IRX6 | Iroquois-class homeodomain protein IRX-6 | -2.76287 | 2.24E-25 | 1.38E-23 |
| ISX | Intestine-specific homeobox | -2.77627 | 2.07E-05 | 6.06E-05 |
| ELF5 | ETS-related transcription factor Elf-5 | -2.7861 | 1.39E-13 | 1.58E-12 |
| OLIG3 | Oligodendrocyte transcription factor 3 | -2.82965 | 0.000107 | 0.000275 |
| SOX8 | Transcription factor SOX-8 | -2.84615 | 4.21E-20 | 1.32E-18 |
| PHOX2B | Paired mesoderm homeobox protein 2B | -2.85895 | 0.000588 | 0.001327 |
| FEZF1 | Fez family zinc finger protein 1 | -2.89871 | 7.26E-10 | 4.52E-09 |
| SP8 | Transcription factor Sp8 | -2.91604 | 0.000701 | 0.00156 |
| ZNF716 | Zinc finger protein 716 | -3.04063 | 3.54E-06 | 1.18E-05 |
| DLX6 | Homeobox protein DLX-6 | -3.05142 | 1.01E-15 | 1.62E-14 |
| FOXA2 | Hepatocyte nuclear factor 3-beta | -3.05711 | 8.90E-09 | 4.64E-08 |
| PAX7 | Paired box protein Pax-7 | -3.11309 | 2.41E-07 | 9.84E-07 |
| DMRT1 | Doublesex- and mab-3-related transcription factor 1 | -3.12936 | 4.04E-13 | 4.24E-12 |
| ESX1 | Homeobox protein ESX1-C | -3.17929 | 2.34E-06 | 8.09E-06 |
| FOXE1 | Forkhead box protein E1 | -3.28562 | 1.27E-13 | 1.46E-12 |
| SPIB | Transcription factor Spi-B | -3.3004 | 1.20E-27 | 9.70E-26 |
| LHX5 | LIM/homeobox protein Lhx5 | -3.31167 | 1.72E-15 | 2.68E-14 |
| ZNF280A | Zinc finger protein 280A | -3.33048 | 2.37E-12 | 2.21E-11 |
| NANOGNB | NANOG neighbor homeobox. | -3.36168 | 3.07E-06 | 1.04E-05 |
| FOSL1 | Fos-related antigen 1 | -3.37426 | 1.89E-50 | 1.05E-47 |
| SOX11 | Transcription factor SOX-11 | -3.58434 | 2.91E-44 | 1.06E-41 |
| CDX2 | Homeobox protein CDX-2 | -3.61693 | 1.46E-13 | 1.64E-12 |
| RAX | Retinal homeobox protein Rx | -3.67625 | 2.99E-18 | 7.20E-17 |
| BARX1 | Homeobox protein BarH-like 1 | -3.70693 | 1.24E-29 | 1.20E-27 |
| FOXG1 | Forkhead box protein G1 | -3.73746 | 3.80E-13 | 4.00E-12 |
| BNC1 | Zinc finger protein basonuclin-1 | -3.80683 | 9.29E-30 | 9.24E-28 |
| HOXD13 | Homeobox protein Hox-D13 | -4.02396 | 2.17E-33 | 3.15E-31 |
| DMRTA2 | Doublesex- and mab-3-related transcription factor A2 | -4.11253 | 9.61E-26 | 6.19E-24 |
| NR2E1 | Nuclear receptor subfamily 2 group E member 1 | -4.31983 | 3.90E-23 | 1.86E-21 |
| PRDM13 | PR domain zinc finger protein 13 | -4.64139 | 5.60E-13 | 5.76E-12 |
| ZIC5 | Zinc finger protein ZIC 5 | -4.66903 | 9.61E-17 | 1.79E-15 |
| ZFP42 | Zinc finger protein 42 homolog | -4.69216 | 1.22E-18 | 3.16E-17 |
| VAX1 | Ventral anterior homeobox 1 | -4.71302 | 1.77E-17 | 3.73E-16 |
| PDX1 | Pancreas/duodenum homeobox protein 1 | -4.99315 | 1.19E-14 | 1.62E-13 |
| POU4F1 | POU domain, class 4, transcription factor 1 | -5.36317 | 7.03E-33 | 9.82E-31 |
| TLX3 | T cell leukemia homeobox 3. | -7.34137 | 2.44E-27 | 1.89E-25 |
| LIN28B | Protein lin-28 homolog B | -7.65309 | 4.87E-30 | 5.03E-28 |

Table s2. Transcription factors in DEGs from differential analysis comparing S100A9-high/low groups (S100A9-high as the reference group)


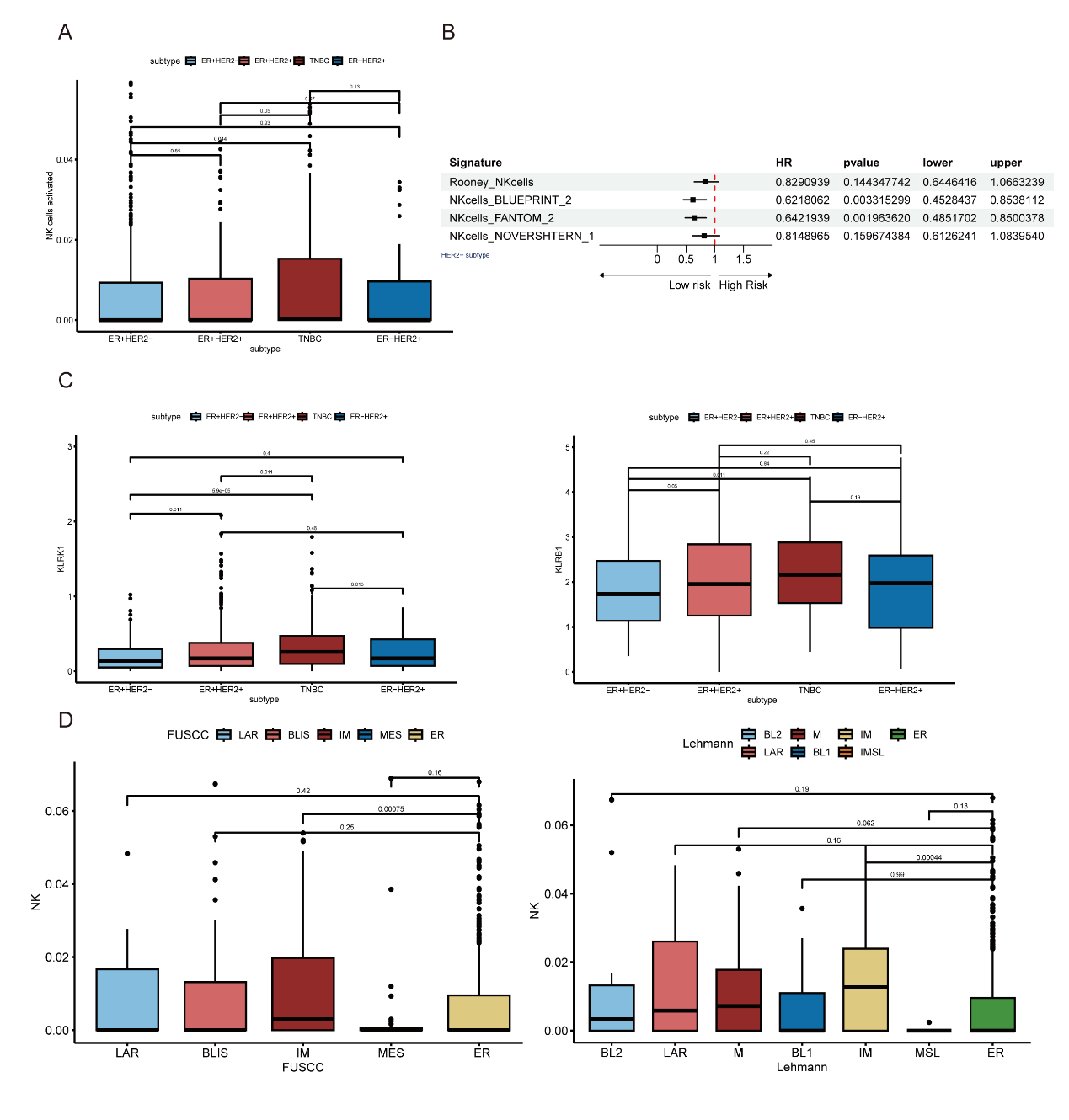


Figure S1

(A) Activated NK cell distributions BC molecular subtypes measured by CIBERSORT. (B) Forest plot of univariate cox regression analyses of NK cell-based signature scores calculated by ssGSEA on HER2+ patients’ survivals. The red dash line represents hazard ratio equals one. (C) Expression levels of NK cell activation markers (KLRB1 and KLRK1) in BC molecular subtypes. (D) Activated NK cell distributions in ER+/HER2- and FUSCC/ Lehmann’s TNBC subtypes measured by CIBERSORT.


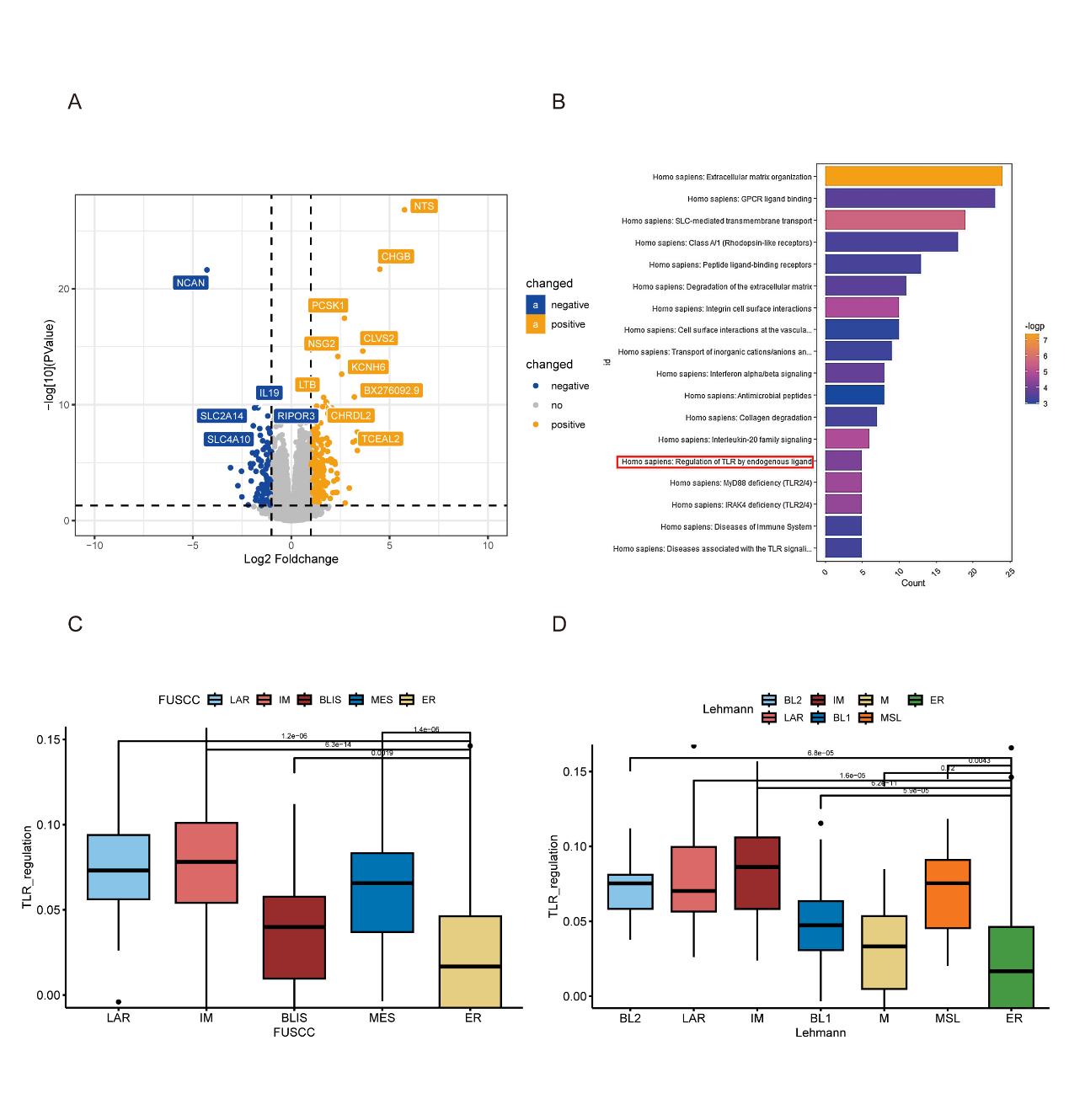


Figure S2

(A)Volcano plot of DEGs between high and low NK cell infiltration samples. (B) Reatome enrichment analysis of differential genes between patients with high or low NK cell levels. (C-D) TLR regulation signature enriched differently in ER+/HER2- and FUSCC/Lehmann’s TNBC subtypes calculated by ssGSEA method.


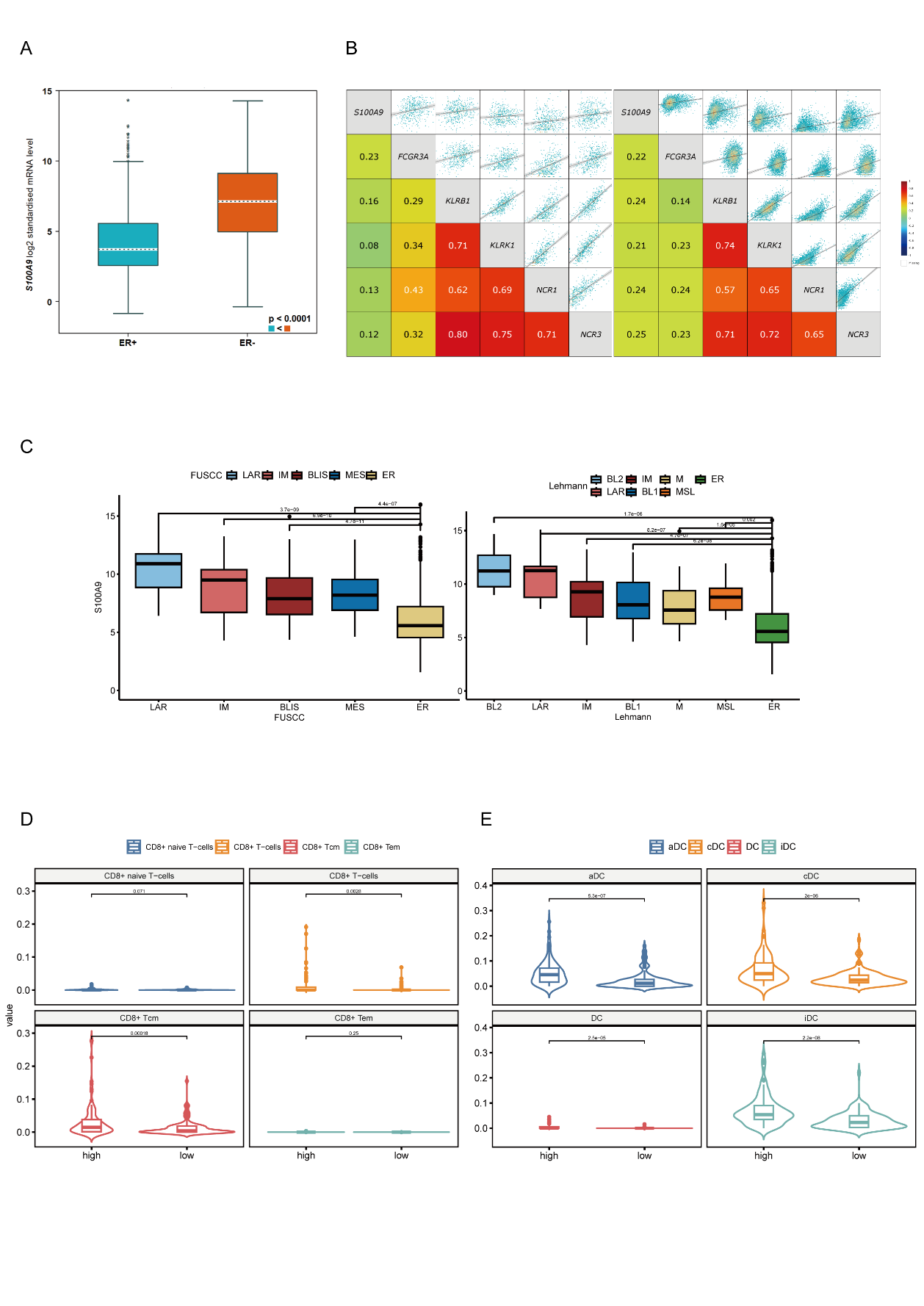


Figure S3

(A) Boxplots of S100A9 mRNA levels of ER+/ER- breast cancer in TCGA cohort. (B) Graphs show the associations of activated NK cell markers and S100A9 expression levels in TNBC (right) and ER+ BC (left). High positive correlations are colored in bright red. (C) Boxplots of S100A9 mRNA levels of ER+/HER2- breast cancer and TNBC subtypes in TCGA cohort. (D-E) Violin plots of the infiltration levels of CD8+ T cell/DC cell and their subtypes quantified by xCell algorithm in S100A9 high/low groups with SCAN-B data.


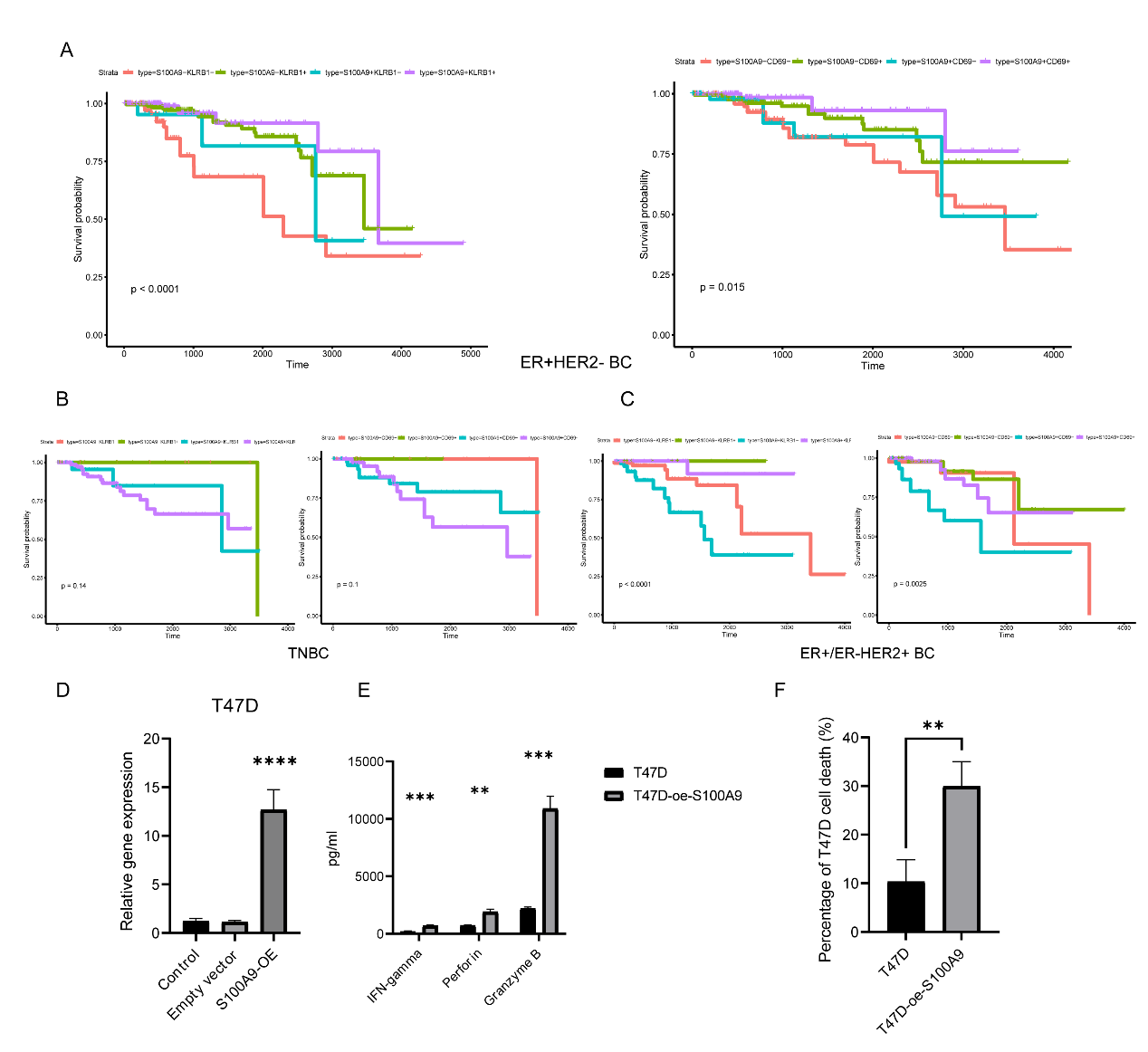


Figure. S4

(A) K-M analyses of OS in ER+/HER2- BC patients stratified by expression levels of S100A9 and KLRB1/ CD69. (B) K-M analyses of OS in TNBC patients stratified by expression levels of S100A9 and KLRB1/ CD69. (C) K-M analyses of OS in ER+/ER-HER2+ BC patients stratified by expression levels of S100A9 and KLRB1/ CD69. (D) The q-PCR experiment validates the S100A9 expression after transfection of S100A9-overexpression vector in T47D cells. (E) ELISA measures the secretion levels of IFN-gamma, perforin and granzyme B from NK cell cocultured with S100A9-overexpressing T47D cell line. (F) LDH NK cell cytotoxicity assay of NK cell cocultured with S100A9-overexpressing T47D cell line. *P < 0.05, **P < 0.01, ***P < 0.001, ****P < 0.0001.
